# Supplementary material for: AnySleep: a channel-agnostic deep learning system for high-resolution sleep staging in multi-center cohorts
Source: NPJ Digit Med. 2026 Oct 30;9:829. doi: 10.1038/s41746-026-03406-7 (PMC13722680; doi:10.1038/s41746-026-03406-7)
Supplement: Supplementary file 1 [file 41746_2026_3406_MOESM1_ESM.pdf]

Supplementary Material

for: AnySleep: a channel-agnostic deep learning system for high-resolution sleep staging in multi-center cohorts

Niklas Grieger[1,2,3], **Jannik Raskob**[1,3], **Siamak Mehrkanoon**[2], **Stephan Bialonski**[1,3]

[1]Department of Medical Engineering and Technomathematics, FH Aachen University of Applied Sciences, 52428 Jülich, Germany, [2]Department of Information and Computing Sciences, Utrecht University, Utrecht, The Netherlands, [3]Institute for Data-Driven Technologies, FH Aachen University of Applied Sciences, 52428 Jülich, Germany

This document contains supplementary figures, tables, and additional experimental details for the manuscript.

Model and code: https://github.com/dslaborg/anysleep

Correspondence: N.G. (grieger@fh-aachen.de), S.B. (bialonski@fh-aachen.de)

S1 Supplementary Analyses and Validation Results

Dataset	N_{Rec}	Wake	N1	N2	N3	REM	MF1	Coh. κ
abc	20	0.90 (.003)	0.60 (.012)	0.84 (.003)	0.76 (.018)	0.91 (.003)	0.80 (.006)	0.77 (.005)
ccshs	78	0.97 (.001)	0.64 (.003)	0.92 (.000)	0.89 (.001)	0.93 (.001)	0.87 (.001)	0.89 (.001)
cfs	92	0.96 (.001)	0.52 (.004)	0.89 (.000)	0.85 (.000)	0.91 (.001)	0.83 (.001)	0.86 (.001)
chat	128	0.96 (.001)	0.65 (.004)	0.87 (.001)	0.91 (.000)	0.91 (.002)	0.86 (.001)	0.86 (.001)
dcsm	39	0.98 (.005)	0.52 (.008)	0.85 (.000)	0.82 (.002)	0.87 (.028)	0.81 (.005)	0.85 (.009)
hpap	36	0.92 (.002)	0.51 (.031)	0.84 (.004)	0.77 (.006)	0.90 (.002)	0.79 (.006)	0.77 (.002)
mesa	100	0.95 (.001)	0.57 (.006)	0.86 (.001)	0.69 (.003)	0.91 (.001)	0.80 (.001)	0.82 (.001)
mros	134	0.96 (.000)	0.45 (.006)	0.87 (.002)	0.71 (.002)	0.89 (.001)	0.78 (.001)	0.83 (.001)
phys	100	0.84 (.006)	0.61 (.010)	0.84 (.002)	0.81 (.002)	0.88 (.002)	0.79 (.002)	0.74 (.001)
sedf-sc	23	0.99 (.000)	0.58 (.005)	0.87 (.002)	0.73 (.017)	0.87 (.015)	0.81 (.004)	0.86 (.002)
sedf-st	8	0.81 (.008)	0.58 (.013)	0.88 (.008)	0.67 (.028)	0.93 (.005)	0.77 (.004)	0.75 (.006)
shhs	140	0.95 (.000)	0.50 (.007)	0.87 (.001)	0.78 (.002)	0.91 (.000)	0.80 (.002)	0.83 (.001)
sof	68	0.96 (.001)	0.46 (.008)	0.86 (.002)	0.77 (.008)	0.92 (.002)	0.79 (.004)	0.83 (.003)
Mean		0.944	0.548	0.869	0.794	0.903	0.812	0.829
dodh	25	0.90 (.005)	0.63 (.022)	0.87 (.017)	0.80 (.022)	0.94 (.004)	0.83 (.012)	0.80 (.017)
dodo	55	0.90 (.000)	0.53 (.016)	0.86 (.013)	0.75 (.022)	0.92 (.002)	0.79 (.008)	0.77 (.012)
isruc-sg1	100	0.89 (.001)	0.51 (.015)	0.80 (.001)	0.80 (.002)	0.88 (.001)	0.78 (.003)	0.73 (.002)
isruc-sg2	16	0.83 (.005)	0.49 (.009)	0.76 (.001)	0.81 (.001)	0.83 (.002)	0.74 (.001)	0.67 (.001)
isruc-sg3	10	0.87 (.010)	0.53 (.011)	0.78 (.005)	0.76 (.017)	0.84 (.010)	0.76 (.007)	0.71 (.009)
mass-c1	53	0.94 (.000)	0.45 (.049)	0.81 (.005)	0.60 (.001)	0.89 (.003)	0.74 (.010)	0.72 (.007)
mass-c3	62	0.92 (.002)	0.57 (.034)	0.86 (.010)	0.74 (.014)	0.91 (.003)	0.80 (.010)	0.77 (.011)
svuh	25	0.81 (.001)	0.33 (.012)	0.81 (.003)	0.85 (.003)	0.87 (.003)	0.74 (.003)	0.70 (.002)
Mean		0.898	0.512	0.825	0.754	0.895	0.777	0.744

Table S1 Detailed performance overview of AnySleep on the in-distribution (upper part of the table) and hold-out test sets (lower part). For each dataset, scores were calculated using all available channels and then weighted by the number of test recordings to obtain weighted mean scores. The model was trained three times with different random seeds, and we report the mean scores and standard deviations (in parentheses).

Dataset	N_{Rec}	Wake	N1	N2	N3	REM	MF1	Coh. κ
abc	20	0.88 (.007)	0.53 (.022)	0.83 (.002)	0.68 (.035)	0.89 (.009)	0.76 (.009)	0.74 (.008)
ccshs	78	0.97 (.001)	0.61 (.008)	0.91 (.002)	0.88 (.001)	0.92 (.003)	0.86 (.003)	0.88 (.003)
cfs	92	0.96 (.002)	0.51 (.007)	0.88 (.002)	0.84 (.004)	0.90 (.006)	0.82 (.004)	0.85 (.003)
chat	128	0.96 (.003)	0.60 (.023)	0.86 (.004)	0.90 (.001)	0.89 (.009)	0.84 (.007)	0.84 (.006)
dcsm	39	0.98 (.001)	0.50 (.016)	0.85 (.003)	0.80 (.011)	0.89 (.009)	0.81 (.005)	0.86 (.004)
hpap	36	0.91 (.002)	0.43 (.009)	0.84 (.003)	0.77 (.003)	0.90 (.004)	0.77 (.002)	0.76 (.002)
mesa	100	0.95 (.001)	0.53 (.024)	0.86 (.001)	0.69 (.008)	0.90 (.002)	0.78 (.006)	0.81 (.003)
mros	134	0.95 (.002)	0.43 (.011)	0.86 (.004)	0.69 (.013)	0.88 (.005)	0.76 (.007)	0.82 (.005)
phys	100	0.84 (.004)	0.57 (.017)	0.84 (.003)	0.81 (.001)	0.88 (.002)	0.79 (.005)	0.74 (.004)
sedf-sc	23	0.98 (.001)	0.56 (.011)	0.86 (.003)	0.75 (.006)	0.86 (.004)	0.80 (.003)	0.84 (.002)
sedf-st	8	0.83 (.011)	0.58 (.013)	0.88 (.008)	0.63 (.003)	0.91 (.009)	0.77 (.005)	0.74 (.014)
shhs	140	0.94 (.005)	0.48 (.010)	0.86 (.003)	0.76 (.009)	0.91 (.002)	0.79 (.005)	0.81 (.006)
sof	68	0.96 (.001)	0.45 (.015)	0.85 (.005)	0.73 (.017)	0.91 (.003)	0.78 (.007)	0.82 (.006)
Mean		0.938	0.517	0.862	0.781	0.896	0.799	0.817
dodh	25	0.88 (.010)	0.60 (.019)	0.86 (.014)	0.79 (.021)	0.93 (.002)	0.81 (.012)	0.79 (.018)
dodo	55	0.89 (.014)	0.51 (.012)	0.87 (.005)	0.77 (.008)	0.93 (.004)	0.79 (.007)	0.78 (.009)
isruc-sg1	100	0.90 (.006)	0.49 (.007)	0.79 (.003)	0.80 (.012)	0.89 (.001)	0.77 (.002)	0.73 (.004)
isruc-sg2	16	0.83 (.002)	0.46 (.005)	0.76 (.002)	0.80 (.011)	0.83 (.001)	0.74 (.002)	0.66 (.005)
isruc-sg3	10	0.89 (.007)	0.56 (.008)	0.78 (.004)	0.77 (.013)	0.86 (.002)	0.77 (.002)	0.72 (.004)
mass-c1	53	0.94 (.002)	0.36 (.027)	0.80 (.007)	0.60 (.013)	0.89 (.001)	0.72 (.009)	0.70 (.007)
mass-c3	62	0.93 (.001)	0.48 (.018)	0.85 (.004)	0.73 (.006)	0.91 (.001)	0.78 (.005)	0.75 (.005)
svuh	25	0.82 (.002)	0.34 (.011)	0.81 (.001)	0.84 (.024)	0.88 (.002)	0.74 (.004)	0.70 (.006)
Mean		0.896	0.469	0.822	0.754	0.898	0.768	0.738

Table S2 Detailed performance overview of U-Sleep on the in-distribution (upper part of the table) and hold-out test sets (lower part). For each dataset, scores were calculated using all available channels and then weighted by the number of test recordings to obtain weighted mean scores. Following Perslev et al. [1], we generated predictions for all EEG-EOG channel pairs and combined them by majority voting. The model was trained three times with different random seeds, and we report the mean scores and standard deviations (in parentheses).

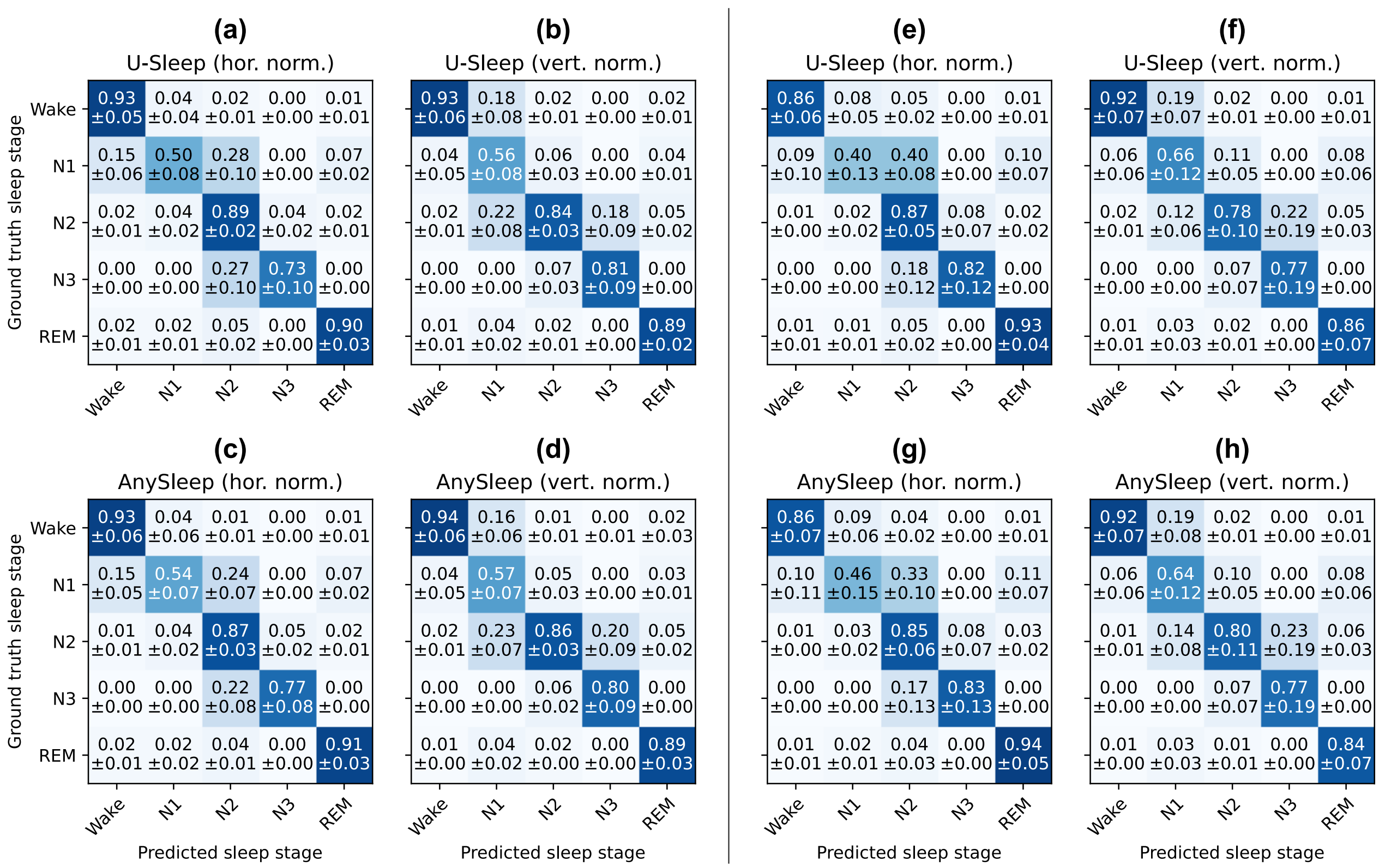

Figure S1 Confusion matrices for U-Sleep (upper panels **(a)**, **(b)**, **(e)**, **(f)**) and AnySleep (lower panels **(c)**, **(d)**, **(g)**, **(h)**) on the test sets. The left panels (**(a)**–**(d)**) show confusion matrices for the in-distribution test sets, while the right panels (**(e)**–**(h)**) show confusion matrices for the hold-out test sets. Matrices were calculated for each dataset and model run, normalized horizontally (panels **(a)**, **(c)**, **(e)**, **(g)**) or vertically (panels **(b)**, **(d)**, **(f)**, **(h)**), and finally averaged (± standard deviation).

Dataset	EEG channels	EOG channels
In-Distribution training and test sets		
abc	F3-M2 (100%), F4-M1 (100%), C3-M2 (100%), C4-M1 (100%), O1-M2 (100%), O2-M1 (100%)	E1-M2 (100%), E2-M1 (100%)
ccshs	C3-A2 (100%), C4-A1 (100%)	LOC-A2 (100%), ROC-A1 (100%)
cfs	C3-A2 (100%), C4-A1 (100%)	LOC-A2 (100%), ROC-A1 (100%)
chat	F3-M2 (100%), F4-M1 (100%), C3-M2 (100%), C4-M1 (100%), T3-M2 (100%), T4-M1 (100%), O1-M2 (100%), O2-M1 (100%)	E1-M2 (100%), E2-M1 (100%)
dcsm	F3-M2 (100%), F4-M1 (100%), C3-M2 (100%), C4-M1 (100%), O1-M2 (100%), O2-M1 (100%)	E1-M2 (100%), E2-M2 (100%)
hpap	F3-M2 (100%), F4-M1 (100%), C3-M2 (100%), C4-M1 (100%), O1-M2 (100%), O2-M1 (100%)	E1-M2 (84%), E2-M1 (85%), E1 (91%), E2 (91%)
mesa	EEG1 [Fz-Cz] (100%), EEG2 [Cz-Oz] (100%), EEG3 [C4-M1] (100%)	EOG-L (100%), EOG-R (100%)
mros	C3-M2 (100%), C4-M1 (100%)	E1-M2 (100%), E2-M1 (100%)
phys	F3-M2 (100%), F4-M1 (100%), C3-M2 (100%), C4-M1 (100%), O1-M2 (100%), O2-M1 (100%)	E1-M2 (100%)
sedf-sc	EEG Fpz-Cz (100%), EEG Pz-Oz (100%)	EOG horizontal (100%)
sedf-st	EEG Fpz-Cz (100%), EEG Pz-Oz (100%)	EOG horizontal (100%)
shhs	EEG [C4-A1] (100%), EEG(sec) [C3-A2] (98%)	EOG(L) (100%), EOG(R) (100%)
sof	C3-A2 (100%), C4-A1 (100%)	LOC-A2 (100%), ROC-A1 (100%)
Hold-Out test sets		
dodh	C3_M2 (100%), F4_M1 (100%), F3_F4 (100%), F3_M2 (100%), F4_O2 (100%), F3_O1 (100%), FP1_F3 (100%), FP1_M2 (100%), FP1_O1 (100%), FP2_F4 (100%), FP2_M1 (100%), FP2_O2 (100%)	EOG1 (100%), EOG2 (100%)
dodo	C4_M1 (100%), C3_M2 (100%), F3_F4 (100%), F3_M2 (100%), F4_O2 (100%), F3_O1 (100%), O1_M2 (100%), O2_M1 (100%)	EOG1 (100%), EOG2 (100%)
isruc-sg1	F3-M2 (100%), F4-M1 (100%), C3-M2 (100%), C4-M1 (100%), O1-M2 (100%), O2-M1 (100%)	E1-M2 (100%), E2-M1 (100%)
isruc-sg2	F3-M2 (100%), F4-M1 (100%), C3-M2 (100%), C4-M1 (100%), O1-M2 (100%), O2-M1 (100%)	E1-M2 (100%), E2-M1 (100%)
isruc-sg3	F3-M2 (100%), F4-M1 (100%), C3-M2 (100%), C4-M1 (100%), O1-M2 (100%), O2-M1 (100%)	E1-M2 (100%), E2-M1 (100%)

mass-c1	EEG F3-CLE (89%), EEG F4-CLE (89%), EEG C3-CLE (89%), EEG C4-CLE (89%), EEG O1-CLE (89%), EEG O2-CLE (89%), EEG F7-CLE (89%), EEG F8-CLE (89%), EEG T3-CLE (89%), EEG T4-CLE (89%), EEG T5-CLE (89%), EEG T6-CLE (89%), EEG P3-CLE (89%), EEG P4-CLE (89%), EEG Fz-CLE (89%), EEG Cz-CLE (89%), EEG Pz-CLE (89%), EEG F3-LER (11%), EEG F4-LER (11%), EEG C3-LER (11%), EEG C4-LER (11%), EEG O1-LER (11%), EEG O2-LER (11%), EEG F7-LER (11%), EEG F8-LER (11%), EEG T3-LER (11%), EEG T4-LER (11%), EEG T5-LER (11%), EEG T6-LER (11%), EEG P3-LER (11%), EEG P4-LER (11%), EEG Fz-LER (11%), EEG Cz-LER (11%), EEG Pz-LER (11%), EEG Fp1-LER (6%), EEG Fp2-LER (6%)	EOG Left Horiz (100%), EOG Right Horiz (100%)
mass-c3	EEG Fp1-LER (100%), EEG Fp2-LER (100%), EEG F7-LER (100%), EEG F8-LER (100%), EEG F3-LER (100%), EEG F4-LER (100%), EEG T3-LER (100%), EEG T4-LER (100%), EEG C3-LER (100%), EEG C4-LER (100%), EEG T5-LER (100%), EEG T6-LER (100%), EEG P3-LER (100%), EEG P4-LER (100%), EEG O1-LER (100%), EEG O2-LER (100%), EEG Fz-LER (100%), EEG Cz-LER (100%), EEG Pz-LER (100%), EEG Oz-LER (100%), EEG A2-LER (69%)	EOG Left Horiz (100%), EOG Right Horiz (100%)
svuh	C3A2 (100%), C4A1 (100%)	Lefteye (100%), RightEye (100%)
	Test sets used for high-frequency evaluations	
cnc	C3-A2 (67%), C3-M2 (23%), C4-A1 (67%), C4-M1 (23%), EEG A1-A2 (5%), EEG C3-A2 (10%), EEG C4-A1 (10%), EEG F3-A2 (10%), EEG F4-A1 (10%), EEG Fp2-A1 (3%), EEG O1-A2 (10%), EEG O2-A1 (10%), F3-A2 (67%), F3-M2 (23%), F4-A1 (67%), F4-M1 (23%), O1-A2 (67%), O1-M2 (23%), O1-O2 (1%), O2-A1 (67%), O2-M1 (23%), O2-O1 (1%)	E1-M2 (23%), E2-M2 (23%), EOG LOC-A2 (10%), EOG ROC-A2 (10%), LOC-A2 (67%), ROC-A1 (67%)
dhc	c3a2 (100%), c4a1 (100%), f3a2 (100%), f4a1 (100%), o1a2 (98%), o2a1 (100%)	eogla2 (100%), eogra1 (100%)
fhc	EEG A1 (62%), EEG A2 (62%), EEG C3 (100%), EEG C4 (100%), EEG Fp1 (38%), EEG Fp2 (38%), EEG O1 (38%), EEG O2 (99%), EEG T3 (38%), EEG T4 (38%), T1 (1%), T2 (1%)	EOG3 (100%), EOGD (100%), EOGG (100%)
ihc	A1 (95%), A2 (95%), C3 (93%), C3/A2 (1%), C4 (95%), C4/A1 (1%), C4_A1 (5%), CZ/A1 (1%), Cz (95%), Cz_A1 (2%), O1 (95%), O1/A2 (1%), O1_A2 (5%), O2_A1 (3%)	EOG_L (100%), EOG_R (100%)
khc	A1 (71%), A2 (71%), C3 (71%), C3-A2 (29%), C4 (71%), C4-A1 (29%), O1 (71%), O1-A2 (29%), O2 (71%), O2-A1 (29%)	EOG Left (71%), EOG Right (71%), EOG-L (29%), EOG-R (29%)
ssc	C3-A1 (24%), C3-A2 (99%), C3-O1 (1%), C4-A1 (99%), C4-A2 (24%), F1-A2 (96%), F2-C4 (24%), F2-T4 (2%), F3-A2 (1%), F4-A1 (1%), FP-? (1%), FP1-C3 (1%), FP2-C4 (1%), Fz-A1 (24%), Fz-A2 (96%), O1-x (99%), O2-x (99%), T3 (1%), T3-O1 (1%), T4-O2 (1%)	L-EOG (2%), LOC (1%), LOC-A2 (97%), R-EOG (2%), ROC (1%), ROC-A1 (97%)

cap	A1 (2%), A2 (2%), C3 (2%), C3-A2 (5%), C3-P3 (65%), C3A2 (3%), C4 (2%), C4-A1 (92%), C4-P4 (92%), C4A1 (3%), F1-F3 (1%), F2-F4 (3%), F3 (2%), F3-C3 (65%), F3A2 (3%), F4 (2%), F4-C4 (92%), F4A1 (3%), F7 (2%), F7-T3 (63%), F8 (2%), F8-T4 (63%), FP1 (2%), FP1-F3 (64%), Fp2 (2%), Fp2-F4 (81%), O1 (2%), O1-A2 (1%), O1A2 (3%), O2 (2%), O2-A1 (4%), O2A1 (3%), P3 (2%), P3-O1 (65%), P4 (2%), P4-O2 (92%), T3 (2%), T3-T5 (42%), T4 (3%), T4-T6 (42%), T5 (2%), T6 (2%)	EOG dx (1%), EOG sin (1%), EOG-L (2%), EOG-R (2%), LOC (4%), LOC / A2 (1%), LOC-A1 (3%), LOC-ROC (3%), ROC (4%), ROC / A1 (1%), ROC-A2 (3%), ROC-LOC (89%)
	High-density EEG test sets	
anphy	Fp1 (100%), Fp2 (100%), F3 (100%), F4 (100%), C3 (100%), C4 (100%), P3 (100%), P4 (100%), O1 (100%), O2 (100%), F7 (100%), F8 (100%), T3 (100%), T4 (100%), T5 (100%), T6 (100%), FZ (100%), CZ (100%), PZ (100%), SO1 (100%), SO2 (100%), F9 (100%), F10 (100%), ZY1 (100%), ZY2 (100%), T9 (100%), T10 (100%), P9 (100%), P10 (100%), AF7 (100%), AF3 (100%), F11 (100%), F5 (100%), F1 (100%), FT11 (100%), FT9 (100%), FT7 (100%), FC5 (100%), FC3 (100%), FC1 (100%), FCZ (100%), C5 (100%), C1 (100%), TP11 (100%), TP9 (100%), TP7 (100%), CP3 (100%), CP1 (100%), P11 (100%), P5 (100%), P1 (100%), PO7 (100%), PO3 (100%), POZ (100%), OZ (100%), FPZ (100%), AFZ (100%), AF4 (100%), AF8 (100%), F2 (100%), F6 (100%), F12 (100%), FC2 (100%), FC4 (100%), FC6 (100%), FT8 (100%), FT10 (100%), FT12 (100%), C6 (100%), C2 (100%), CPZ (100%), CP2 (100%), CP4 (100%), CP6 (100%), TP8 (100%), TP10 (100%), TP12 (100%), P2 (100%), P6 (100%), P12 (100%), PO4 (100%), PO8 (100%), CP5 (100%)	EOG1 (100%), EOG2 (100%)

Table S3 EEG and EOG channels available in each dataset. Datasets are grouped by their role in the study. Percentages refer to the proportion of recordings in which each channel was available. Where not self-evident, electrode configurations are given in square brackets (LER = linked-ear reference, CLE = computed linked-ear [2]).

Metric	Expert	AnySleep	Δ to expert	ρ_{Spear}	U-Sleep	Δ to expert
TST	6:17±1:14	6:23±1:13	-0:06±0:19	0.97	6:23±1:13	-0:06±0:18
Wake duration	1:30±0:59	1:24±0:57	0:06±0:19	0.95	1:23±0:56	0:06±0:18
N1 duration	0:50±0:29	0:35±0:25	0:15±0:29	0.50	0:29±0:21	0:21±0:28
N2 duration	3:12±1:07	3:18±0:49	-0:05±0:47	0.69	3:27±0:50	-0:15±0:46
N3 duration	1:05±0:40	1:13±0:36	-0:08±0:36	0.55	1:12±0:37	-0:07±0:36
REM duration	1:09±0:32	1:16±0:34	-0:07±0:13	0.93	1:14±0:34	-0:05±0:13
Sleep efficiency	78.80±14.06%	82.38±13.27%	-3.57±3.57%	0.96	81.50±13.38%	-2.70±3.47%
WASO	1:16±0:50	1:01±0:47	0:15±0:21	0.96	1:04±0:46	0:12±0:20
REM latency	2:06±1:01	2:05±1:07	0:00±0:39	0.87	2:11±1:09	-0:05±0:43
Sleep latency	0:18±0:20	0:17±0:24	0:01±0:19	0.91	0:18±0:24	0:00±0:17

Table S4 Sleep metrics derived from expert annotations and predicted hypnograms from AnySleep and U-Sleep for the hold-out datasets at 30-s resolution. Metrics are reported as mean±standard deviation of hours:minutes across recordings, with Δ values and Spearman's ρ quantifying recording-wise differences (calculated as expert−model) and associations between expert-derived and prediction-derived metrics, respectively. Total sleep time (TST) and sleep-specific durations were obtained by summing the duration of the respective stages. Sleep efficiency, wake after sleep onset (WASO), REM sleep latency, and sleep onset latency were calculated solely for the ISRUC datasets, the only hold-out datasets with available lights-off/on times required by the AASM [3] guidelines. We note that the ISRUC datasets consist of both healthy controls and patients with various disorders including sleep apnea, REM sleep disorder, and affective disorders.

Model	N_{Rec}	Wake	N1	N2	N3	REM	MF1	Coh. κ
AnySleep	28	0.91 (.002)	0.46 (.009)	0.90 (.000)	0.91 (.005)	0.88 (.002)	0.81 (.002)	0.82 (.002)
U-Sleep	28	0.89 (.010)	0.47 (.001)	0.89 (.005)	0.87 (.019)	0.87 (.002)	0.80 (.004)	0.79 (.006)

Table S5 Performance of AnySleep and U-Sleep on the high-density held-out ANPHY [4] dataset. This dataset contains 28 recordings from 13 female and 15 male healthy subjects (mean age 32.46 ± 6.10 years). Scores were calculated using the 83 EEG and 2 EOG channels contained in each recording (see Supplementary Table S3). For U-Sleep's majority voting approach, this required 166 evaluations of different EEG-EOG channel pairs. The models were trained three times with different random seeds, and we report the mean scores and standard deviations (in parentheses).

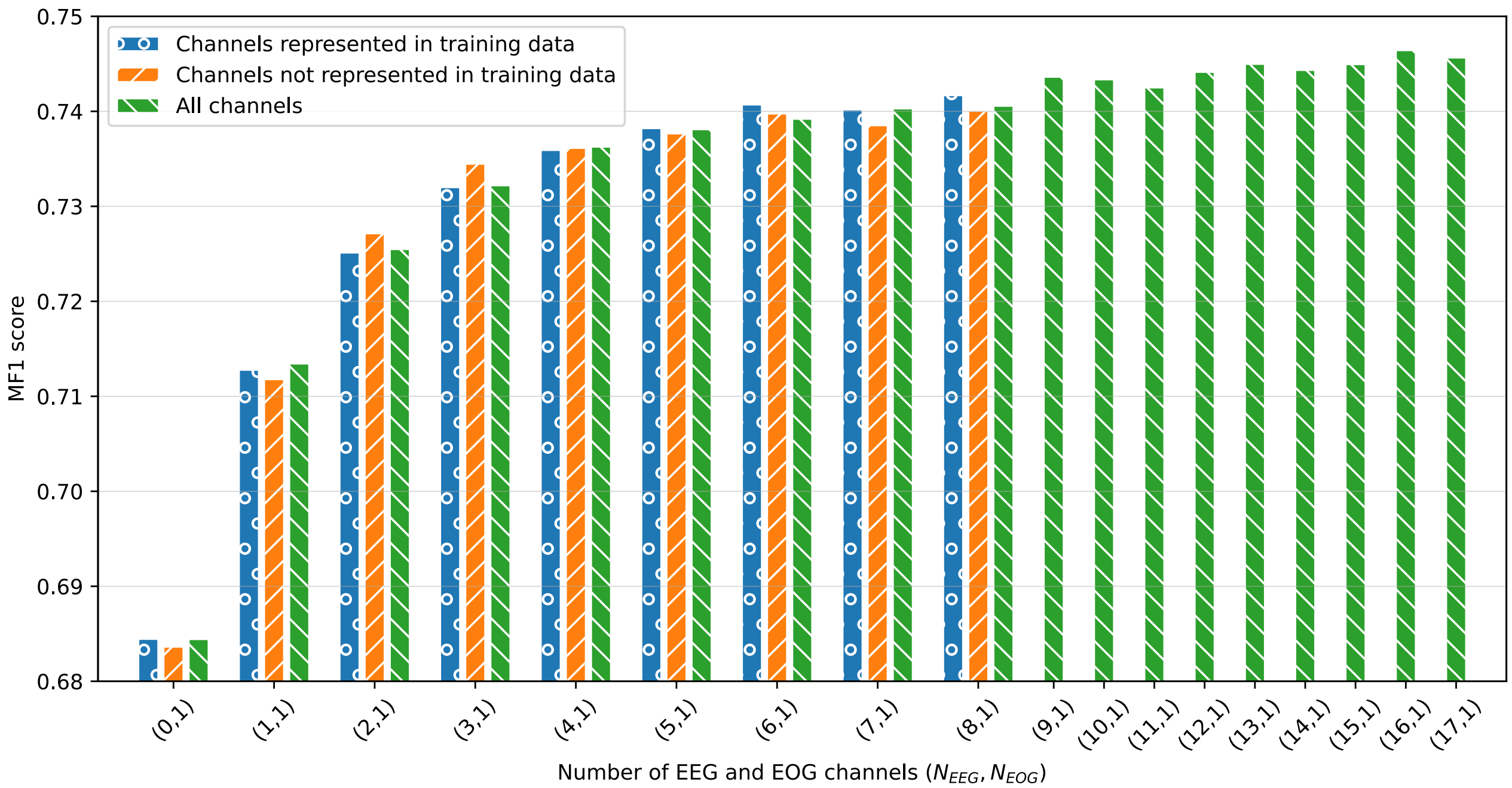

Figure S2 Generalization of AnySleep to unseen channels across varying channel counts. AnySleep was evaluated on the mass-c1 and mass-c3 datasets with one fixed EOG channel and three EEG configurations: (i) only EEG derivations used during training (blue), (ii) only EEG channels not used during training (orange), and (iii) all available EEG channels (green). Performance is reported as recording-wise macro F1 scores and grouped by the combination of EEG (N_{EEG}) and EOG (N_{EOG}) channels. For each channel-count condition, we randomly sampled 5000 recordings with replacement and evaluated each on a randomly chosen subset of channels of that size (without replacement). Evaluations were repeated for three independent training runs, and we report the average scores over recordings and runs. Model performance increased with the number of channels across all conditions, with no substantial difference between seen and unseen channels, indicating that AnySleep generalizes to previously unseen channels without performance degradation.

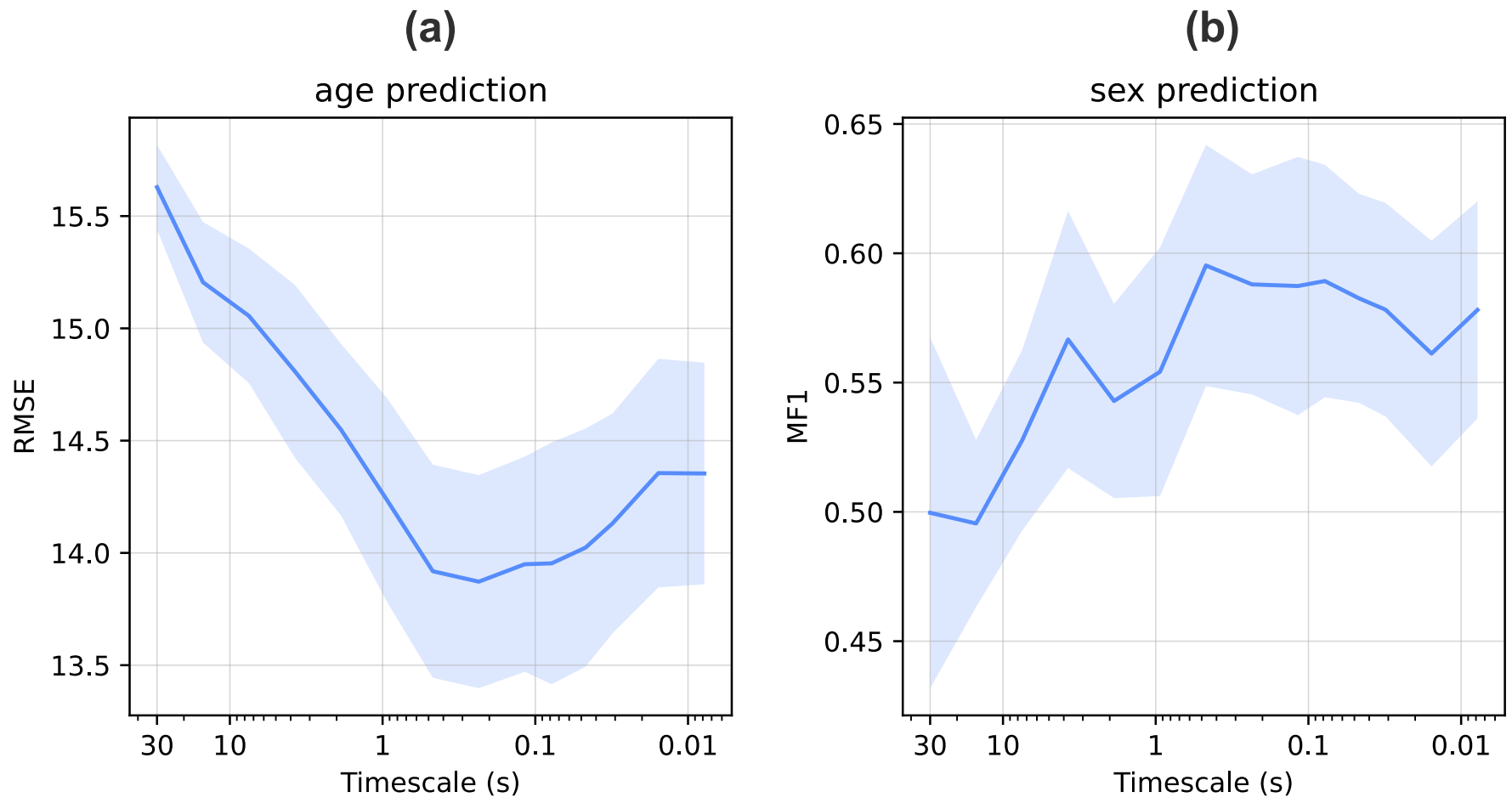

Figure S3 Prediction of physiological characteristics (age, sex) from triplet features derived from AnySleep's high-frequency sleep stages at different timescales on held-out datasets. **(a)** Root mean squared error (RMSE) of a random forest (RF) regression model predicting age from triplet features for 116 subjects from ISRUC sg1–3; recording-wise age predictions were calculated as the average of predictions across 1.5 h blocks (see Section 4.5). **(b)** Macro F1 (MF1) scores of an RF classifier predicting sex (male vs female) for 118 subjects from ISRUC sg1–3. For each timescale and task in (a) and (b), 50 RF models were trained on features derived from each of three independent AnySleep training runs (150 RF models in total); the blue line and blue shaded area show mean score and standard deviation across these models. Model performance improved as the timescale of the high-frequency sleep stages decreased, suggesting that AnySleep's high-frequency sleep stages contain additional age- and sex-associated information compared to 30-s sleep stages.

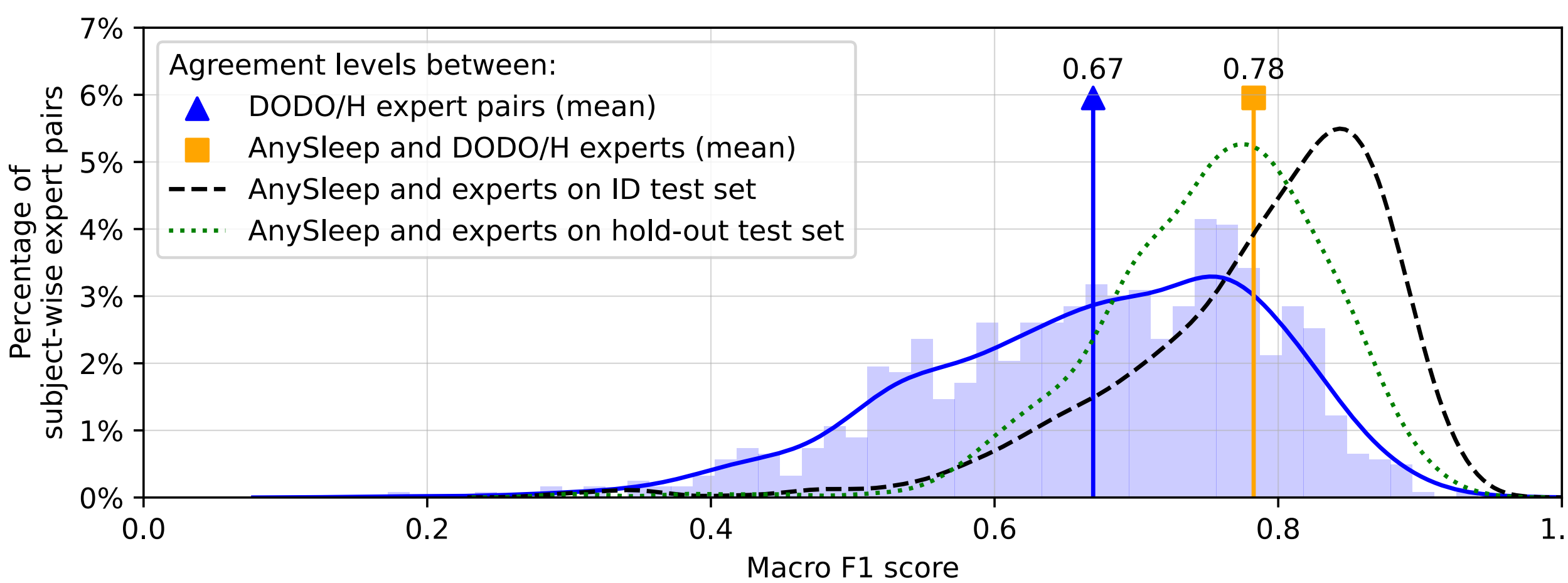

Figure S4 Comparison between AnySleep-expert and expert-expert agreement on held-out DODO and DODH datasets. Blue bars show a histogram of agreements levels (measured by MF1 scores) between pairs of experts for each subject in the combined DODO/H datasets, which were annotated by six different experts. We also show a density estimate of the expert agreement distribution (blue curve) and the average expert-expert agreement level (blue vertical line). AnySleep-expert agreement was calculated subject-wise for each available expert annotation and aggregated across the three training runs, with the average agreement level shown in orange. For context, density estimates show the distributions of AnySleep-expert agreement across all in-distribution test datasets (black dashed curve) and hold-out test datasets (green dotted curve). AnySleep-expert agreement on DODO/DODH was comparable to or higher than typical expert-expert agreement, indicating that the model performs within the range of human inter-rater variability on these datasets.

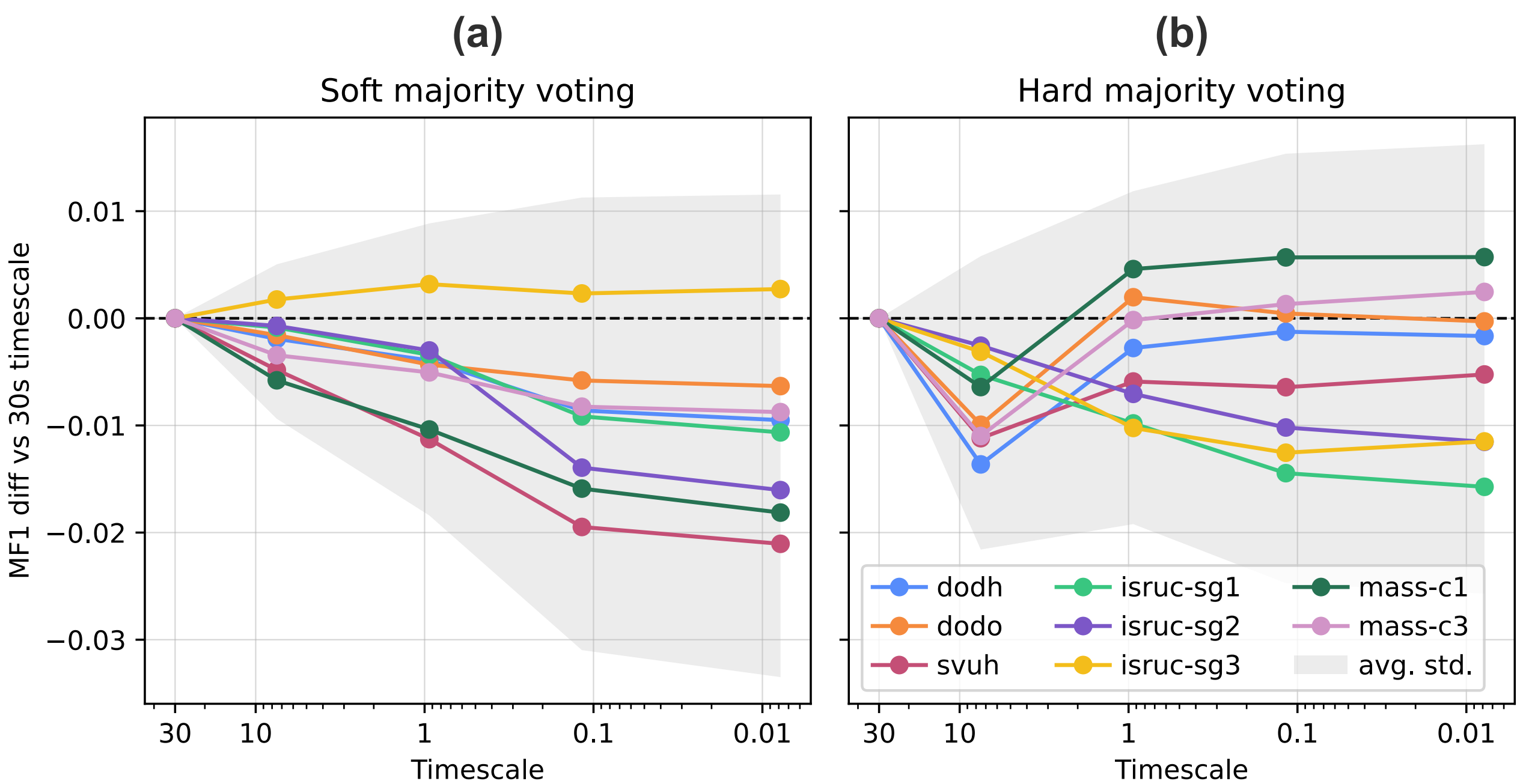

Figure S5 Comparison of approaches for aggregating AnySleep's high-frequency representations into 30-s labels on the hold-out test datasets. Each curve plots the difference in macro F1 (MF1) between AnySleep evaluated at a given timescale and evaluations at the 30-s baseline. At the 30-s timescale, internal high-frequency representations are aggregated with an average pooling layer in the segment classifier (see Fig. 5c). At lower timescales, the internal high-frequency representations are first downsampled into the respective timescale by the average pooling layer, passed through the remainder of the segment classifier, and then aggregated into 30-s labels using either soft majority voting (panel **(a)**; average of output logits over the sub-intervals of each 30-s window) or hard majority voting (panel **(b)**; majority count of predicted sleep stages with ties broken at random). MF1 differences were calculated for each subject and each of three training runs. We report the average MF1 difference over subjects and training runs for each hold-out test dataset, as well as the average standard deviation (avg. std.) across datasets. Results varied by dataset, as soft majority voting largely reduced MF1 except for isruc-sg3 while hard majority voting improved MF1 for some datasets such as mass-c1 but not for others.

References

[1] Perslev, M. *et al.* U-Sleep: Resilient high-frequency sleep staging. *npj Digit. Med.* **4**, DOI: https://doi.org/10.1038/S41746-021-00440-5 (2021).

[2] O'Reilly, C., Gosselin, N., Carrier, J. & Nielsen, T. Montreal Archive of Sleep Studies: An open-access resource for instrument benchmarking and exploratory research. *J. Sleep Res.* **23**, 628–635, DOI: https://doi.org/10.1111/jsr.12169 (2014).

[3] Berry, R. B. *et al. The AASM manual for the scoring of sleep and associated events: Rules, terminology and technical specifications, Version 2.6* (American Academy of Sleep Medicine, Darien, Illinois, 2020).

[4] Wei, X. *et al.* ANPHY-Sleep: an Open Sleep Database from Healthy Adults Using High-Density Scalp Electroencephalogram. *Sci. Data* **11**, DOI: https://doi.org/10.1038/s41597-024-03722-1 (2024).
